# Prevalence and influencing factors of pandemic fatigue among Chinese public in Xi'an city during COVID-19 new normal: a cross-sectional study

**DOI:** 10.3389/fpubh.2022.971115

**Published:** 2022-09-12

**Authors:** Ling Xin, Liuhui Wang, Xuan Cao, Yingnan Tian, Yisi Yang, Kexin Wang, Zheng Kang, Miaomiao Zhao, Chengcheng Feng, Xinyu Wang, Nana Luo, Huan Liu, Qunhong Wu

**Affiliations:** ^1^Department of Health Policy, School of Health Management, Harbin Medical University, Harbin, China; ^2^Department of Social Medicine, School of Public Health, Harbin Medical University, Harbin, China; ^3^School of Business and Economics, University of San Carlos, Cebu, Philippines; ^4^Harbin Center for Disease Control and Prevention, Harbin, China; ^5^Department of Health Management, School of Public Health, Nantong University, Nantong, Jiangsu, China; ^6^Department of Critical Care Medicine, The First Affiliated Hospital of Harbin Medical University, Harbin, China

**Keywords:** COVID-19, new normal, pandemic fatigue, public, influencing factors

## Abstract

**Objective:**

This study aimed to assess Chinese public pandemic fatigue and potential influencing factors using an appropriate tool and provide suggestions to relieve this fatigue.

**Methods:**

This study used a stratified sampling method by age and region and conducted a cross-sectional questionnaire survey of citizens in Xi'an, China, from January to February 2022. A total of 1500 participants completed the questionnaire, which collected data on demographics, health status, coronavirus disease 2019 (COVID-19) stressors, pandemic fatigue, COVID-19 fear, COVID-19 anxiety, personal resiliency, social support, community resilience, and knowledge, attitude, and practice toward COVID-19. Ultimately, 1354 valid questionnaires were collected, with a response rate of 90.0%. A binary logistic regression model was used to examine associations between pandemic fatigue and various factors.

**Result:**

Nearly half of the participants reported pandemic fatigue, the major manifestation of which was “being sick of hearing about COVID-19” (3.353 ± 1.954). The logistic regression model indicated that COVID-19 fear (OR = 2.392, 95% CI = 1.804–3.172), sex (OR = 1.377, 95% CI = 1.077–1.761), the pandemic's impact on employment (OR = 1.161, 95% CI = 1.016–1.327), and COVID-19 anxiety (OR = 1.030, 95% CI = 1.010–1.051) were positively associated with pandemic fatigue. Conversely, COVID-19 knowledge (OR = 0.894, 95% CI = 0.837–0.956), COVID-19 attitude (OR = 0.866, 95% CI = 0.827–0.907), COVID-19 practice (OR = 0.943, 95% CI = 0.914–0.972), community resiliency (OR = 0.978, 95% CI = 0.958–0.999), and health status (OR = 0.982, 95% CI = 0.971–0.992) were negatively associated with pandemic fatigue.

**Conclusion:**

The prevalence of pandemic fatigue among the Chinese public was prominent. COVID-19 fear and COVID-19 attitude were the strongest risk factors and protective factors, respectively. These results indicated that the government should carefully utilize multi-channel promotion of anti-pandemic policies and knowledge.

## Introduction

The corona virus disease 2019 (COVID-19) pandemic has caused approximately 500 million infections and 6 million deaths worldwide by June 2022 (1), and the global pandemic is ongoing. As many countries have adopted the strategy of coexisting with the virus, COVID-19 constantly mutates, creating what has been termed the new normal characterized by dynamic changes and repeated invasion of COVID-19 (2). During the new normal, China has adopted a dynamic zero-COVID policy, which has achieved remarkable results. However, during the new normal with vaccines and medicine that cannot obtain further breakthroughs, achieving zero infection remains challenging (3). According to data reported by National Health Commission, the new wave of COVID-19 has spread to 30 provinces in China and infected nearly 500,000 people from March to mid-April 2022 (4). One study noted that the global incidence rate of mental health problems has risen sharply due to the COVID-19 pandemic (5). Due to the constant risk of COVID-19 infection, public mental health deserves attention.

Fatigue may be the most noticeable among numerous mental health problems. A poll showed that 75% of participants felt fatigued by the COVID-19 pandemic (6). In addition, the feeling of fatigue may affect public compliance with infection prevention and control policies, including refusing to wear a mask or seek information (7). In October 2020, World Health Organization (WHO) introduced the concept of pandemic fatigue, which refers to the demotivation to adhere to protective behavior (8). Subsequently, scholars used other similar terms, such as quarantine fatigue (9) and behavioral fatigue (10); however, pandemic fatigue was considered the most appropriate concept (11, 12). Nevertheless, there is no consensus on the concept, existence, and scope of application of pandemic fatigue in academia (13). Some scholars have questioned the validity of pandemic fatigue and argued that there is little evidence of fatigue influencing compliance with infection prevention and control among the public (10, 11). Therefore, it is particularly important to verify and assess pandemic fatigue.

This new concept has attracted significant attention, and Google's search volume on the concept was over 240 million at the start of 2021 (14). However, few have addressed this concept, with most studies being reviews (11, 14, 15) and scarce quantitative research. A study in Turkey used an ordinary fatigue scale to assess the fatigue status of 3672 people and found that 64.1% of participants had fatigue problems during the pandemic (16). MacIntyre et al. examined the presence of pandemic fatigue by comparing changes in the number of infection prevention measures taken by participants during two specific periods and found that participants' infection pandemic prevention and control practices consistently decreased, indicating pandemic fatigue (17). Although some scholars have tried to quantify pandemic fatigue, few studies have developed and used specific tools to assess public pandemic fatigue (18). In addition, studies on pandemic fatigue in China have been scarce, and the development and usage of local measurement tools are lacking.

Potential influencing factors of pandemic fatigue include COVID-19 fear (12), younger age (17), anxiety due to COVID-19 (16), and COVID-19-related knowledge, attitude, and practice (KAP) (16). However, analyses of potential influencing factors of pandemic fatigue remain few and dispersed. In the context of the new normal, with the progression of the pandemic and development and changes in prevention and control policies, it is particularly important to explore the potential influencing factors of public pandemic fatigue in China.

This study aimed to assess pandemic fatigue in China and its influencing factors using an appropriate tool. The results are expected to provide guidelines to relieve public pandemic fatigue and improve the efficiency of infection prevention and control.

## Materials and methods

### Study setting and data collection

In late 2021, the new wave of COVID-19 in Xi'an, Shaanxi, became the next serious issue since the outbreak of Wuhan. It was in this region and period that we collected data for this study: using a cross-sectional questionnaire, we surveyed citizens in Xi'an from January to February 2022. The sample size was calculated based on the following formula:


$$\begin{array}{l} N={\left(\frac{Z_{\frac{1-a}{2}}}{\delta }\right)}^{2}\times p\times \left(1-p\right) \end{array}$$

where Z_1−α/2_ = 1.96 (α = 0.05), δ represented the margin of error, which was 0.03, and p was the value closest to 50% of the possible sample rate. The minimal sample size calculated by this formula was roughly 1062. To reduce sampling error and improve study quality, 1500 questionnaires were collected.

**Figure 1:**
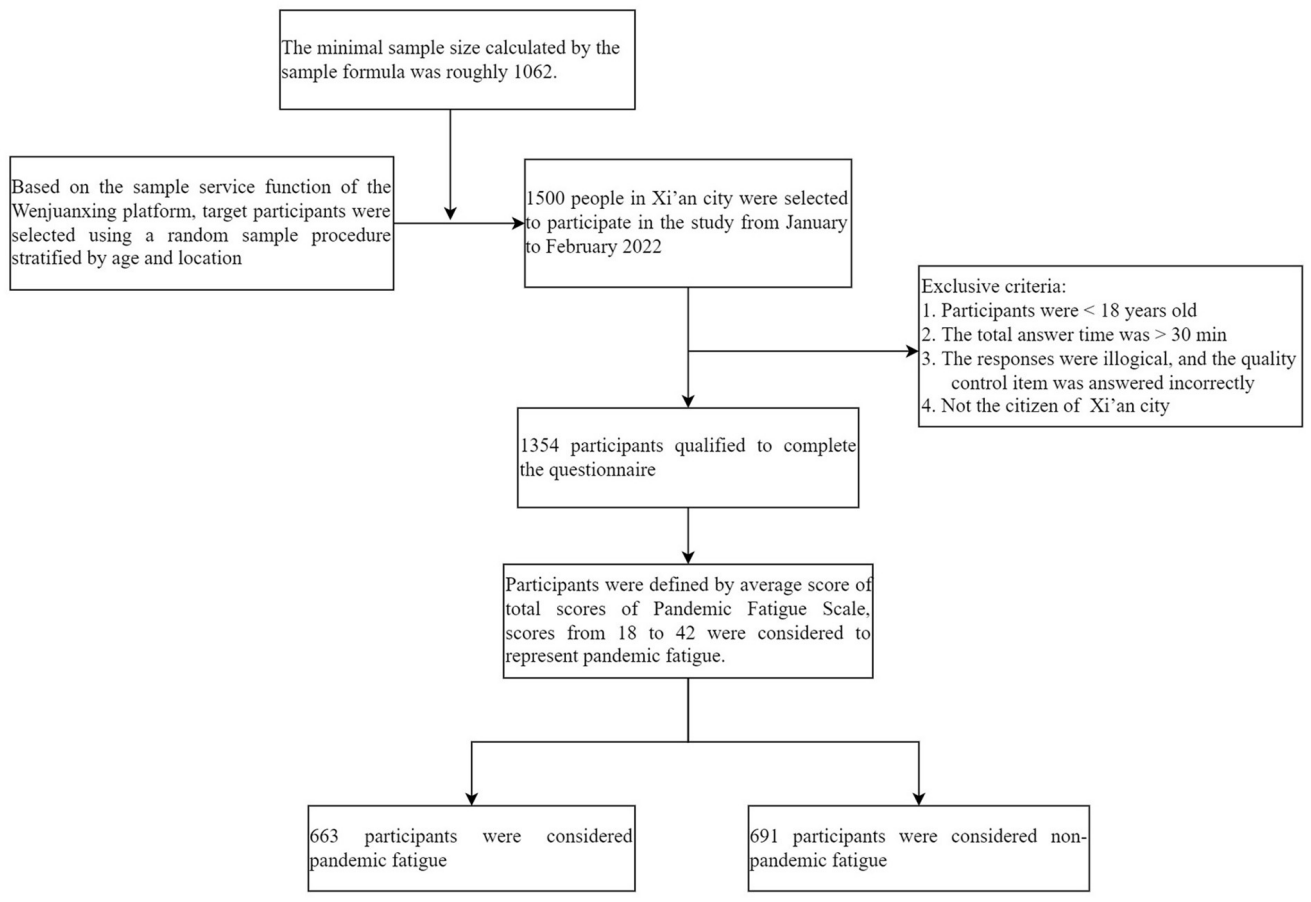
Flowchart of participants inclusion and exclusion.

Considering the timeliness and feasibility of the survey, the sample service function of the Wenjuanxing platform was used to conduct this online survey and collect questionnaires. The Wenjuanxing platform is the largest online survey platform in China, whose database contains over 2 million representative, diverse, and authentic sample populations. Based on the sample service function of the Wenjuanxing platform, target participants were selected using a random sample procedure stratified by age and location from the Wenjuanxing sample database. The questionnaire link was sent to participants via the Wenjuanxing platform, and participants could use mobile phones, computers, or tablets to complete the questionnaire online. All data was made available for viewing and downloading on the Wenjuanxing platform. Questionnaires were considered valid if participants were ≥ 18 years old, the total answer time was ≤ 30 min, the responses were logical, the quality control item was answered correctly, and participants were the citizen of Xi'an. According to these inclusion criteria, two researchers respectively performed the procedure for cleaning up data and excluding invalid questionnaires to check the validity of the answers. Only valid responses were included in the study. A total of 1354 questionnaires were retained, with a recovery rate of 90.0% (for details, see Figure 1).

### Ethics approval statement

This study was approved by the Ethics Review Board of Harbin Medical University. Participation was voluntary, and the anonymity of the respondents was maintained. Informed consent was provided by the respondents online before completing the questionnaire (HMUIRB20200003).

### Variables

A structured questionnaire was designed to obtain demographic and social-economic characteristics, health status, COVID-19 stressors, pandemic fatigue, COVID-19 anxiety, COVID-19 fear, personal resiliency, social support, community resiliency, and KAP towards COVID-19.

#### Dependent variable

The dependent (outcome) variable was pandemic fatigue. The six-item Pandemic Fatigue Scale (PFS) in English was originally developed by Lilleholt et al. to measure individuals' fatigue from COVID-19 (7). To ensure “linguistic and conceptual equivalence” (19), translation into Chinese and back-translation into English were conducted. The participant rated the agreement with each item on a seven-point Likert scale (1 = strongly disagree; 7 = strongly agree). The PFS yields a total fatigue score ranging from 6 to 42, and higher total scores indicate more severe fatigue. Based on the average score in the present study, scores ranging from 18 to 42 were considered to represent pandemic fatigue. The original PFS has good internal consistency, strong content validity, excellent concurrent validity, and well-established discriminant validity. In the present study, the PFS exhibited acceptable internal consistency (alpha coefficient = 0.848, McDonald's omega = 0.849).

#### Independent variable

Demographic and socio-economic characteristics included age, gender, employment status, education background, personal monthly income, marital status, and the number of children.

Ahorsu et al. developed the Fear of COVID-19 Scale (FCV-19S) (20) to measure individuals' fear levels about COVID-19. We used Chinese version FCV-19S developed by Feng et al. (21). The scale comprised seven items rated on a five-point Likert scale (1 = strongly disagree; 5 = strongly agree). Total scores ranged from 7 to 35, with higher scores indicating a higher level of fear. For fear, scores from 7 to 15 were considered low; scores from 16 to 35 were considered high (21). Cronbach's α and McDonald's omega of this scale were 0.86 and 0.859 in our study, respectively, which indicated the scale has good reliability.

We used the Chinese version five-level EuroQol five-dimensional questionnaire (EQ-5D-5L) to assess participants' health status (22). EQ-5D-5L comprises five dimensions: mobility, self-care, usual activities, pain/discomfort, and anxiety/depression. Each dimension was rated on a five-point Likert scale (1 = no problems; 5 = extreme problems). An EQ-5D-5L Value Set for China was used to calculate utility scores reflecting health status (23, 24). Utility scores range from negative values, which indicate a condition worse than death, to 1, representing perfect health, where 0 is defined as death (25). The EQ-5D-5L showed acceptable internal consistency (alpha coefficient = 0. 740, McDonald's omega = 0.756) in our study

We used the COVID-19 Anxiety Syndrome Scale (C19-ASS), comprising nine items for assessing features of COVID-19-related anxiety (26). The Chinese C19-ASS was developed by translators proficient in English and simplified Chinese using standardized translation and back translation procedures. The participants were asked to rate the extent to which each statement applied to them over the last 9 weeks using a five-point Likert scale (0 = not at all; 4 = nearly every day). Scores ranged from 0 to 36. Higher scores are associated with higher levels of anxiety. In this study, the C19-ASS scale showed good reliability (Cronbach's α = 0.838, McDonald's omega = 0.840), consistent with previous studies (27).

Following Crandall et al. (28), we used three questions to explore the participants' COVID-19 stress. First, participants were asked whether they were close contacts or patients using binary responses (1 = yes; 0 = no). In addition, we asked participants whether their employment or income had been affected by COVID-19; responses were rated on a five-point Likert scale (1 = very little; 5 = significantly).

Zimet et al. developed the Multidimensional Scale of Perceived Social Support (MSPSS) to measure respondents' social support from three dimensions: family, friends, and other important people (29). We used the Chinese version MSPSS to measure social support (30). The scale was scored on a seven-point Likert scale (1 = very strongly disagree; 7 = very strongly agree). Higher total scores indicated higher social support. Low social support was defined as a score from 12 to 48, moderate social support was defined as a score from 49 to 68, and scores from 69 to 84 indicated high social support (29). In this study, the MSPSS scale had excellent reliability (Cronbach's α = 0.895, McDonald's omega = 0.896).

The 10-item Connor-Davidson Resilience Scale (CD-RISC-10.) was developed by Campbell to examine the individual's ability to cope with disaster (31). In addition, the CD-RISC-10 applies to various populations and countries (32, 33). We used the Chinese version CD-RISC-10 (34). The scale comprised 10 items and was rated based on how the respondent felt during the past month using a five-point Likert scale (0 = not at all; 4 = almost always). The total scores ranged from 0 to 40, with higher scores representing greater resilience. In the present study, the CD-RISC-10 scale showed good reliability (Cronbach's α = 0.858, McDonald's Omega = 0.859), similar to previous studies (35).

Community resilience was measured using the 10-Item Conjoint Community Resiliency Assessment Measurement (CCRAM-10), which was used to assess the ability of the community to cope with adversity (36). The Chinese CCRAM-10 was developed by translators proficient in English and simplified Chinese using standardized translation and back translation procedures. Participants were asked about their extent of agreement, which they rated on a five-point Likert scale (1 = strongly disagree; 5 = strongly agree). Scores ranged from 10 to 50. Higher scores were associated with greater community resilience. The CCRAM-10 showed good reliability (Cronbach's α = 0.898, McDonald's Omega = 0.899) in our study, consistent with a previous study (37).

Based on the latest Diagnosis and Treatment Protocol for Novel Coronavirus Pneumonia (Trial Version 8, Revised Version) during our study (38), this study designed the questionnaire to assess respondents' KAP levels regarding the prevention and control of COVID-19 during the past month. A 16-item test was applied to evaluate their knowledge of infection prevention and control. Correct responses were recorded as 1 point; incorrect responses and answers of “do not know” were recorded as 0. The scores of 16 items were added to yield the knowledge dimension, with scores ranging from 0 to 16, and higher scores indicated better knowledge. The attitude dimension consisted of seven items rated on a five-point Likert scale (1 = strongly disagree; 5 = strongly agree). The scoring was reversed for the negative attitude items. The scoring system of attitude ranged from 7 to 35, with higher scores indicating a more positive attitude. In the dimension of practice, scores were calculated based on participants' responses to 11 items, rated on a five-point Likert scale (1 = never; 5 = always). Practice dimension scores ranged from 11 to 55 (after reverse-scoring the negative practice items), with a higher score indicating better compliance with infection prevention and control. The scores of the three dimensions were added to yield a total score ranging from 18 to 106, with higher scores indicating greater KAP levels. Following Guo et al., we classified the scores of each dimension of KAP and the total scores. Scores > full marks ×85% were defined as “good and higher than good”; scores ≤ full marks ×85% indicated “lower than good” (39). The questionnaire showed acceptable internal consistency (alpha coefficient = 0.779, McDonald's Omega = 0.784) in our study.

### Statistical analyses

After deleting invalid questionnaires (*n* = 146), 1354 valid questionnaires were recovered for data analysis. Descriptive statistics were calculated, including frequencies and percentages for binary/categorical variables and means and standard deviations for normally distributed continuous variables. In addition, we used median and interquartile ranges to describe continuous variables that were not normally distributed. Pearson's chi-squared test and *t*-tests were used to perform univariate analysis, and binary logistic regression was used to examine associations between pandemic fatigue and its associated factors. All data were analyzed using SPSS 21.0, and *p* < 0.05 was considered significant.

## Results

### Participant characteristics

General participant characteristics are shown in Table 1. As this study adopted an online survey form, most of the respondents were young, with an average age of 32 years. Within the total sample, over half were women (54.1%), were married or cohabitating (60.5%), held a bachelor's degree or above (68.2%), were employed (73.1%), and had children (54.3%). Nearly half of the respondents (46.1%) had a monthly income of <5000 yuan. Pandemic fatigue was reported by 49% of participants. The univariate analysis indicated that gender, COVID-19 fear, social support, knowledge score, attitude score, practice score, being in close contact with a patient or being a patient, the pandemic's impact on employment, the pandemic's impact on income, COVID-19 anxiety, community resilience, personal resilience, and health status were all significantly associated with pandemic fatigue (*p* < 0.05).

**Table 1:** Characteristics of questionnaire respondents and univariate analysis results.

**Variable**	**Category**	**Total *n* (%)**	**Pandemic fatigue**		***P*-value^c^**
			**Yes *n* = 663 (%)**	**No *n* = 691 (%)**	
Age (years)^a^	≤ 24	310 (22.9)	151 (22.8)	159 (23.0)	0.349
	25–30	363 (26.8)	183 (27.6)	180 (26.0)	
	31–36	327 (24.2)	169 (25.5)	158 (22.9)	
	≥37	354 (26.1)	160 (24.1)	194 (28.1)	
Sex^a^	Men	621 (45.9)	338 (51.0)	395 (57.2)	0.025
	Women	733 (54.1)	325 (49.0)	296 (42.8)	
Educational background^a^	Junior college or below	430 (31.8)	219 (33.0)	211 (30.5)	0.350
	Bachelor degree or above	924 (68.2)	444 (67.0)	480 (69.5)	
Occupation^a^	Unemployed, retired or others	364 (26.9)	187 (28.2)	177 (25.6)	0.297
	Employed	990 (73.1)	476 (71.8)	514 (74.4)	
Marital status^a^	Married or cohabitating	819 (60.5)	397 (59.9)	422 (61.1)	0.657
	Unmarried, divorced, or widowed	535 (39.5)	266 (40.1)	269 (38.9)	
Have children^a^	Yes	735 (54.3)	306 (46.2)	313 (45.3)	0.785
	No	619 (45.7)	357 (53.8)	378 (54.7)	
Monthly salary (yuan)^a^	<1000	181 (13.4)	95 (13.7)	86 (13.0)	0.981
	1000–2999	167 (12.3)	85 (12.3)	82 (12.4)	
	3000–4999	276 (20.4)	142 (20.5)	134 (20.2)	
	5000–7999	396 (29.2)	203 (29.4)	193 (29.1)	
	≥8000	334 (24.7)	166 (24.0)	168 (25.3)	
COVID-19 fear^a^	Low	498 (36.8)	154 (23.2)	344 (49.8)	<0.001
	High	856 (63.2)	509 (76.8)	347 (50.2)	
Social support^a^	Low	240 (17.7)	135 (20.4)	105 (15.2)	<0.001
	Medium	788 (58.2)	400 (60.3)	388 (56.2)	
	High	326 (24.1)	128 (19.3)	198 (28.7)	
Knowledge score^b^		13.28 ± 2.16	12.82 ± 2.55	13.72 ± 1.59	<0.001
Attitude score^b^		30.28 ± 3.31	29.28 ± 3.57	31.24 ± 2.71	<0.001
Practice score^b^		48.23 ± 4.86	46.94 ± 5.40	49.46 ± 3.90	<0.001
Be in close contact with a patient or be a patient^a^	Yes	560 (41.4)	293 (44.2)	267 (38.6)	<0.041
	No	794 (58.6)	370 (55.8)	424 (61.4)	
The pandemic's impact on employment^a^	Very small	62 (4.6)	17 (2.6)	45 (6.5)	<0.001
	Relatively small	138 (10.2)	56 (8.4)	82 (11.9)	
	General	399 (29.5)	167 (25.2)	232 (33.6)	
	Relatively large	516 (38.1)	294 (44.3)	222 (32.1)	
	Very large	239 (17.6)	129 (19.5)	110 (15.9)	
The pandemic's impact on income^a^	Very small	99 (7.3)	37 (5.6)	62 (9.0)	<0.001
	Relatively small	194 (14.3)	97 (14.6)	97 (14.0)	
	General	485 (35.8)	214 (32.3)	271 (39.2)	
	Relatively large	375 (27.7)	195 (29.4)	180 (26.0)	
	Very large	201 (14.8)	120 (18.1)	81 (11.7)	
COVID-19 anxiety^b^		19.65 ±7.24	20.56 ± 6.66	18.78 ± 7.67	<0.001
Community resilience^b^		37.62 ± 7.08	36.33 ± 6.81	38.84 ± 7.12	<0.001
Personal resiliency^b^		27.22 ± 6.22	26.32 ± 5.92	28.09 ± 6.38	<0.001
Health state^b^		89.31 ± 14.84	86.0 ± 17.28	92.45 ± 11.18	<0.001

a Data are presented as number (%);

b Data are presented as mean ± standard deviation;

c *p*-value was calculated using the *t*-test and chi-square test.

### Score analysis of pandemic fatigue scale

**Figure 2:**
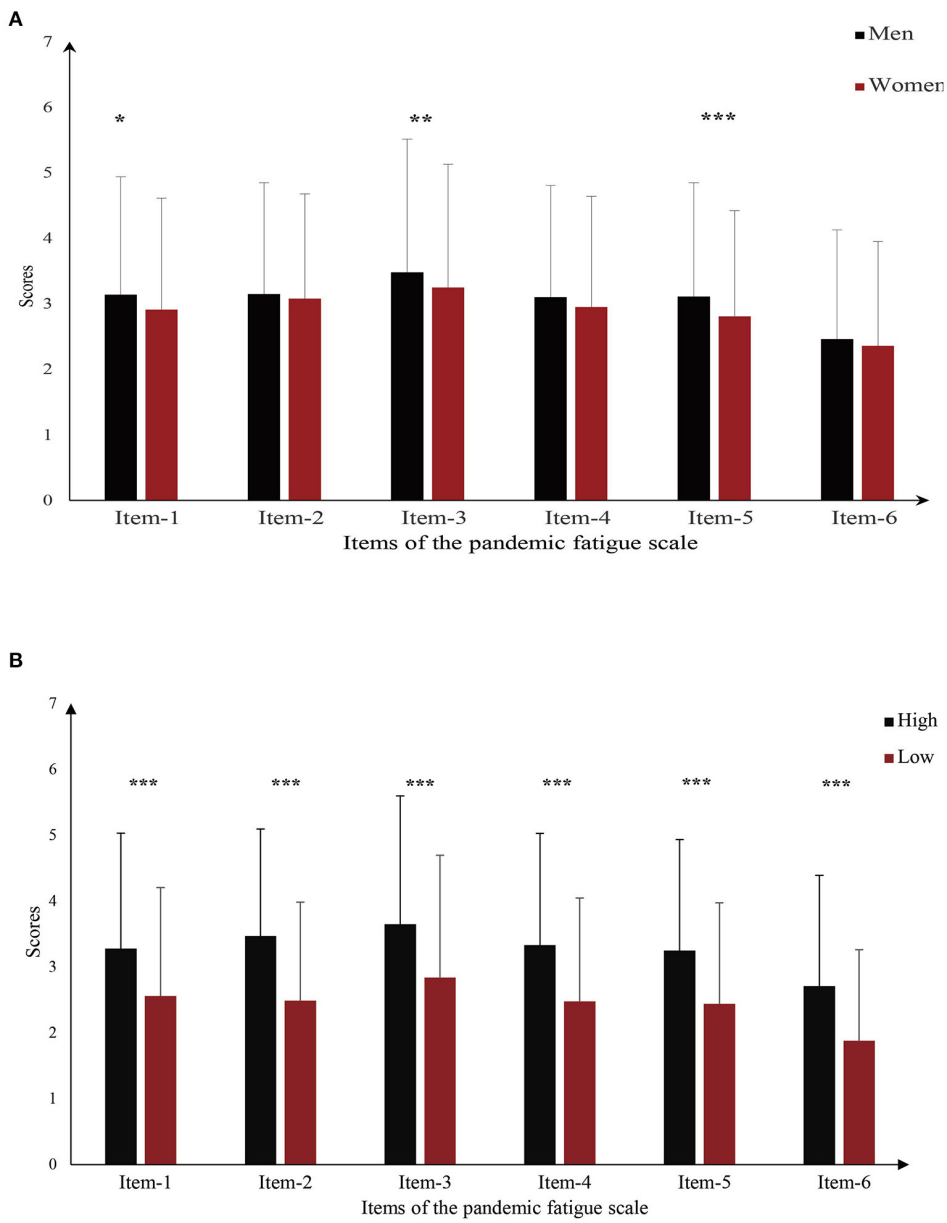
Relationship between pandemic fatigue items and other variables. **(A)** Gender differences in pandemic fatigue item scores; **(B)** Differences in pandemic fatigue items scores for different COVID-19 fear levels. *p < 0.05, **p < 0.01, ***p < 0.001.

Table 2 shows the items of Pandemic Fatigue Scale, which we ranked based on the mean values for each item. “Being sick of hearing about COVID-19” was a major manifestation associated with pandemic fatigue (3.353 ± 1.954), followed by “feeling strained from following all of the behavioral regulations and recommendations around COVID-19” (3.110 ± 1.646). In addition, “being tired of all the COVID-19 discussions in TV shows, newspapers, and radio programs” (3.017 ± 1.749) was a key manifestation. Furthermore, differences in PFS mean scores for six specific items between genders were measured (Figure 2A). The result revealed differences in scores for items 1, 3, and 5 (*p* < 0.05). Figure 2B shows the relationship between specific items of pandemic fatigue and COVID-19 fear. The results demonstrate that, among all pandemic fatigue items, scores of each item were significantly higher for respondents with high-level COVID-19 fear than those with low-level COVID-19 fear.

**Table 2:** Pandemic fatigue items and rank ordering.

**Items number**	**Items**	**Mean ±standard deviation**	**Rank**
1	I am tired of all the COVID-19 discussions in TV shows, newspapers, and radio programs, etc.	3.017 ± 1.749	3
2	I am sick of hearing about COVID-19.	3.353 ± 1.954	1
3	When friends or family members talk about COVID-19, I try to change the subject because I do not want to talk about it anymore.	2.948 ± 1.677	5
4	I feel strained from following all of the behavioral regulations and recommendations around COVID-19.	3.110 ± 1.646	2
5	I am tired of restraining myself to save those who are most vulnerable to COVID-19.	3.016 ± 1.699	4
6	I am losing my spirit to fight against COVID-19.	2.405 ± 1.627	6

Rank based on mean value.

### KAP questionnaire score analysis

**Figure 3:**
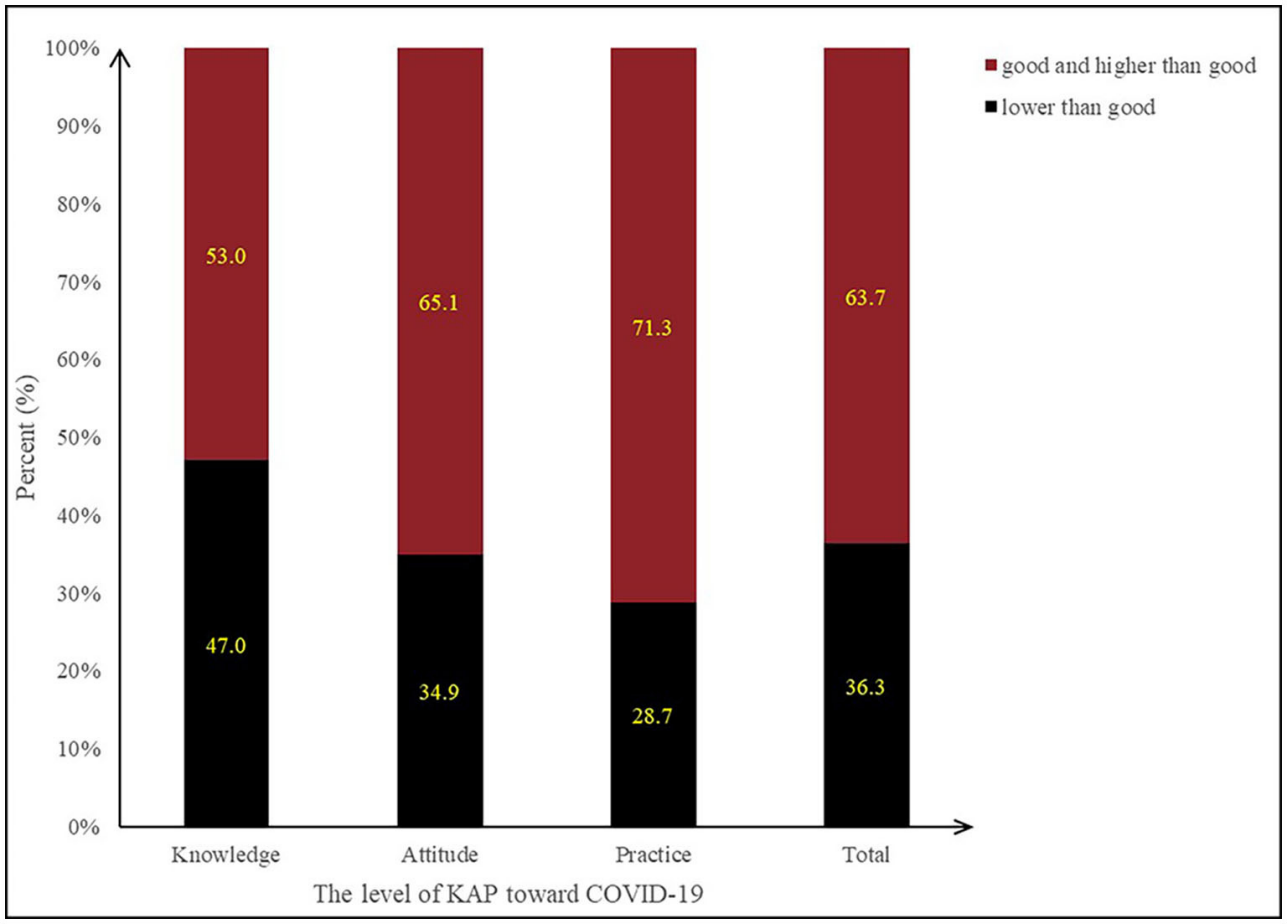
Participants' total KAP levels toward COVID-19 and levels for the three KAP dimensions.

Figure 3 shows the participants' overall COVID-related KAP level and levels of each of its three dimensions (knowledge, attitude, and practice). Nearly 40% of participants had KAP scores lower than “good.” Among the scores for the three dimensions, COVID-19 knowledge was the lowest, followed by attitude level; practice level scores were the highest among the three. We ranked items comprising the attitude and practice dimensions by their mean values (for details, see Supplementary Table 2 and Table 3). For attitude, “Do you discriminate or ostracize COVID-19 patients or the communities in which they live?” had the lowest mean score (3.71 ± 1.23), followed by “Are you currently actively paying attention to the COVID-19 situation at home and abroad?” (3.99 ± 0.85). The third lowest item was “Do you agree that China can successfully prevent infection from imported strains?” (4.31 ± 0.83). The three lowest means scores in the practice dimension, from lowest to third-lowest, were “Do you immediately disinfect the packages after collecting them?” (3.45 ± 1.27), “Do you actively participate in community infection prevention and control and provide suggestions for infection prevention and control?” (3.89 ± 1.10), and “Do you wash your hands after coming home?” (4.21 ± 0.97).

**Table 3:** Factors associated with COVID-19 pandemic fatigue among participants based on the multivariate analysis.

**Variable**	**β**	**Standard error**	**Wald**	**Odds ratio (95% confidence interval)**	***P*-value**
Sex	0.320	0.125	6.526	1.377 (1.077, 1.761)	0.011
COVID-19 fear	0.872	0.144	36.720	2.392 (1.804, 3.172)	<0.001
Social support	– 0.106	0.116	0.834	0.899 (0.716, 1.129)	0.361
Knowledge score	– 0.112	0.034	10.873	0.894 (0.837, 0.956)	0.001
Attitude score	– 0.144	0.023	37.792	0.866 (0.827, 0.907)	<0.001
Practice score	– 0.059	0.016	13.811	0.943 (0.914, 0.972)	<0.001
Be in close contact with a patient or be a patient	0.051	0.104	0.237	1.052 (0.858, 1.290)	0.626
The pandemic's impact on employment	0.149	0.068	4.778	1.161 (1.016, 1.327)	0.029
The pandemic's impact on income	– 0.004	0.063	0.005	0.996 (0.880, 1.126)	0.943
COVID-19 anxiety	0.030	0.010	8.541	1.030 (1.010, 1.051)	0.003
Community resilience	– 0.022	0.011	4.006	0.978 (0.958, 0.999)	0.045
Personal resiliency	– 0.003	0.012	0.073	0.997 (0.973, 1.021)	0.787
Health state	– 0.019	0.005	12.511	0.982 (0.971, 0.992)	<0.001

### Factors associated with pandemic fatigue based on the logistic regression model

Independent variables that were significant predictors of pandemic fatigue in the chi-square tests and *t*-tests were entered into the logistic regression analysis model. The results showed that pandemic fatigue had an inverse association with community resilience (OR = 0.978, 95% CI = 0.958–0.999) and health state (OR = 0.982, 95% CI = 0.971–0.992). Higher knowledge scores (OR = 0.894, 95% CI = 0.837–0.956), attitude scores (OR = 0.866, 95% CI = 0.827–0.907), and practice scores (OR = 0.943, 95% CI = 0.914–0.972) reduced the odds of pandemic fatigue. Compared with the respondents with low levels of COVID-19 fear, respondents with high COVID-19 fear were 2.392 times (95% CI = 1.804–3.172) more likely to suffer from pandemic fatigue. Other significant risk factors included the pandemic's impact on employment (OR = 1.161, 95% CI = 1.016–1.327) and COVID-19 anxiety (OR = 1.030, 95% CI = 1.010–1.051). Furthermore, men were more likely than women to report pandemic fatigue (OR = 1.377, 95% CI = 1.077–1.761).

## Discussion

This study revealed that pandemic fatigue was common in Xi'an. In addition, the results indicated that potential influencing factors of pandemic fatigue included COVID-19 fear, gender, the pandemic's impact on employment, COVID-19 anxiety, KAP towards COVID-19, community resilience, and health status. These study findings clarifying factors of pandemic fatigue could be used as a reference and as a basis for relieving public pandemic fatigue and enhancing public participation and cooperation in infection prevention and control policies.

Our study also showed that COVID-19 fear was the strongest risk factor associated with pandemic fatigue. There are two potential reasons for this. On the one hand, the result revealed that individuals with high COVID-19 fear tended to avoid information related to COVID-19, which was the major manifestation of pandemic fatigue in our study. Avoiding information may create difficulty in analyzing and judging information regarding COVID-19, thereby weakening the motivation to observe infection prevention measures. On the other hand, COVID-19 fear may cause several mental health problems (40), further aggravating pandemic fatigue. Therefore, the government and the department of health should provide psychological assistance to relieve public COVID-19 fear and carefully promote information related to COVID-19 through multiple channels (41) to reduce the likelihood of pandemic fatigue.

A higher impact of the pandemic on employment was associated with a higher level of pandemic fatigue. Approximately 60% of participants reported their employment had been significantly affected by the COVID-19 pandemic. In fact, during this study, Xi'an experienced the most serious COVID-19 outbreak thus far since the first in Wuhan; in response, the government rapidly implemented lockdown measures to prevent further the spread of the pandemic. The lockdown measures may have affected regular employment, particularly flexible employment, which accounts for nearly 30% of total employment in China (42, 43). Moreover, many workers were forced to telecommute, and a survey in China indicated that remote work might reduce work efficiency and cause emotional exhaustion (44), both of which could result in pandemic fatigue. Although the dynamic zero-COVID policy minimizes infection prevention and control's impacts on public life and employment, policymakers should focus on stabilizing public employment, especially flexible employment.

Participants who were more anxious about COVID-19 than average were more likely to experience pandemic fatigue, consistent with a previous study (16). Another study in China indicated that the detection rate of anxiety was over 50% among all participants during COVID-19 (45). Anxious individuals may feel highly nervous and sensitive to COVID-19; these conditions could lead to further pandemic fatigue. Although the pandemic was predicted to end in early 2022 (46), the global pandemic is ongoing (1), and the Chinese public has to face repeated threats from COVID-19. Consequently, public mental health is also a growing concern, and policymakers should take action, including implementing advanced prevention and control measures, such as establishing additional mental assistance hotlines to alleviate public COVID-19 anxiety. During the COVID-19 outbreak in Xi'an in early 2022, psychology experts reportedly served over 1300 people from Xi'an and other lockdown areas in 1 week, providing psychological counseling that helped relieve people's anxiety and promote adherence to anti-infection recommendations (47).

This study used a self-designed questionnaire to assess the level of KAP regarding COVID-19 and found a positive correlation between KAP toward COVID-19 and pandemic fatigue. Participants with higher levels of KAP tended to have sufficient understanding of COVID-19 and confidence in coping with the pandemic; thus, they were motivated to observe infection prevention measures (48). However, we also identified some prominent vulnerabilities. First, approximately 60% of the study participants had only a vague understanding of China's dynamic zero-COVID policy (for details, see Supplementary Table 1 and Supplementary Figure 1). Thus, they may have doubts about the effectiveness of the strategy and the anti-infection measures; such doubts may contribute to fatigue regarding the ongoing pandemic. Second, more than 50% of participants thought of COVID-19 as a “big flu,” which may lead them to underestimate the hazards of COVID-19 and let their guard down (49), further refusing to follow infection prevention recommendations. However, it is noteworthy that because the government realized that the public might have only a vague understanding of anti-pandemic policy due to its short implementation, some experts were invited to publicize and explain the policy to the public through official media (49). This effort to inform the public critically improved public understanding and support of current COVID-19 prevention and control measures, and it should help relieve pandemic fatigue (16).

For the attitude dimension, discrimination and stigma in COVID-19 patients were the most prominent. Similarly, a cross-sectional study in Lebanon suggested that more than half of the respondents had discrimination against COVID-19 patients (50). The increase in discrimination and stigmatization may be due to individuals' fear of COVID-19 infection (50). Significantly, the latest research points out that the discharged patients with COVID-19 are safe in life and work (51). Thus, policymakers should intensify publicity of COVID-19-related information and encourage the public to better understand COVID-19. Meanwhile, the government also must pay attention to COVID-19 patients' mental health to avoid psychological distress and burdens (52). Another important issue in the practice dimension is the non-disinfection of collected packages. Wang and his colleagues indicated that severe acute respiratory syndrome coronavirus 2 (SARS-CoV-2) can survive on the contaminated surface of packaging materials (53). Aiming to address this problem, in addition to the frequent disinfection of the package by the express companies, the government should also have posters reminding the public to disinfect their packages in a timely manner at the express station.

Community resilience is crucial to relieving pandemic fatigue. Resilient communities can better cope with and recover quickly from disasters (54). Individuals living in a highly resilient community can access several resources, including effective leadership, access to services, and good infrastructure (36). These resources greatly increased the public's sense of security and facilitated quick action to cope with COVID-19 (55). Significantly, the COVID-19 pandemic provides a “key opportunity” to construct community resilience. During COVID-19 the government actively promotes infection prevention resources to communities (56) and advances the improvement of community prevention and control systems. However, communities have weaknesses, including insufficient reserves of public health resources (57), imperfect community participation systems, and so on (58). Policymakers should attach importance to the construction of community resilience and enhance residents' sense of belonging and security in the community, which would help relieve public pandemic fatigue.

There was a negative correlation between health status and pandemic fatigue. COVID-19 pandemic increases the strain on the healthcare system and causes a drain on medical resources (59, 60). People in poor health may be unable to access the medical resources they need in time. In addition, the implementation of non-pharmaceutical interventions may make it difficult for the public to conveniently access health services during the pandemic (61). Lacking timely access to medical resources may increase COVID-19 fear among people in poor health (62), thereby causing pandemic fatigue. Although the government adopted a series of measures to ensure a sufficient supply of medical resources, there are still shortages in regions where the pandemic has been severe (63). In the future, the health management department should take further steps to avoid drains on medical resources and help relieve pandemic fatigue.

In addition, gender was the only significant factor associated with pandemic fatigue among the demographic variables. Men were approximately 1.3 times more likely to experience pandemic fatigue than women. Compared to women, men were more likely to feel tired of discussion about COVID-19, avoid talking about COVID-19, and feel fatigued from non-pharmaceutical interventions. This may be because men were less likely to perceive COVID-19 as a serious health problem (64) and, therefore, reluctant to comply with recommendations.

## Limitations

This study had some limitations. First, as this study was cross-sectional, it could not establish causality. Second, this study used online questionnaires and self-reported data, which may result in a reporting bias. Third, there may be differences in the feeling of pandemic fatigue between infected and non-infected people with COVID-19. However, only less than 1% of the participants were infected from COVID-19 in this study, which may have little impact on the results. To assess the prevalence of pandemic fatigue among the public, representative participants were selected from the lockdown area of Xi'an city and specific fatigue scale was used. Therefore, the study results reflect the real level of pandemic fatigue of Xi'an citizens during lockdown. Forth, the results may not reflect the general situation of public fatigue in China due to the study being conducted only in Xi'an. A larger scope of investigation and sample size are required to reveal the panorama of public pandemic fatigue in the future.

## Conclusion

Although the dynamic zero-COVID policy created a marked effect on pandemic prevention, the prevalence of pandemic fatigue among the Chinese public was high. COVID-19 fear was the strongest risk factor associated with pandemic fatigue, whereas COVID-19 attitude was the strongest protective factor. In the new normal, pandemic fatigue deserves further attention. The government should implement measures to address public mental health problems and further reduce the impact of pandemic prevention and control on public employment and healthcare. Furthermore, policymakers should promote information related to COVID-19 through multiple channels and strengthen the control of false information. Moreover, COVID-19 provides a “key opportunity” to construct community resilience, and the government should pay further attention to the construction of community resilience.

## Data availability statement

The raw data supporting the conclusions of this article will be made available by the authors, without undue reservation.

## Ethics statement

The studies involving human participants were reviewed and approved by the Ethics Review Board of Harbin Medical University (HMUIRB20200003). The patients/participants provided their written informed consent to participate in this study.

## Author contributions

HL and LX drafted the manuscript. XC and LW designed the questionnaire and analyzed data. QW and HL designed, organized, and conducted the study, and serve as guarantors for the manuscript. YT, CF, and YY involved in the collection of resources to help write the manuscript. KW and ZK revised the paper. MZ, XW, and NL participated in the literature review. All authors contributed to the article and approved the submitted version.

## Funding

This study was funded by the Innovative Science Research Fund of Harbin Medical University (also known as Heilongjiang Provincial University's Project of Graduate Scientific Research Business Fees) and the Postdoctoral Program of Heilongjiang Province (LBH-Z21181). This work was also supported by the Social Science Foundation of Nantong (2020CNT010).

## Conflict of interest

The authors declare that the research was conducted in the absence of any commercial or financial relationships that could be construed as a potential conflict of interest.

## Publisher's note

All claims expressed in this article are solely those of the authors and do not necessarily represent those of their affiliated organizations, or those of the publisher, the editors and the reviewers. Any product that may be evaluated in this article, or claim that may be made by its manufacturer, is not guaranteed or endorsed by the publisher.

## Abbreviations

COVID-19: corona virus disease 2019
KAP, knowledge, attitude: and practice
WHO: World Health Organization
SARS-CoV-2: severe acute respiratory syndrome coronavirus 2.
