# Supplementary material for: Prevalence and influencing factors of pandemic fatigue among Chinese public in Xi'an city during COVID-19 new normal: a cross-sectional study
Source: Front Public Health. 2022 Sep 12;10:971115. doi: 10.3389/fpubh.2022.971115 (PMC9511105; doi:10.3389/fpubh.2022.971115)
Supplement: Supplementary file 1 [file Data_Sheet_1.pdf]

Supplementary Material

1 Supplementary Tables

Supplementary Table 1. Questionnaire to evaluate knowledge, attitude, and practice among participants regarding COVID-19

COVID-19 knowledge items	Answers
**Item 1:** Transmission route of COVID-19 is:	① Droplet respiratory particles<br>② Air<br>③ Contact goods contaminated by coronavirus<br>④ All of the above
**Item 2:** With the mutation of coronavirus, the infectivity of current epidemic strains has increased.	①yes<br>②no<br>③I do not know
**Item 3:** Children, the elderly, and people with chronic diseases are susceptible to COVID-19.	①yes<br>②no<br>③I do not know
**Item 4:** Patients with symptoms of COVID-19 infection mostly showed flu-like symptoms, such as headache, sore throat, runny nose, fever, and persistent cough.	①yes<br>②no<br>③I do not know
**Item 5:** COVID-19 is "influenza major."	①yes<br>②no<br>③I do not know
**Item 6:** Patients infected with COVID-19 could have no obvious clinical symptoms.	①yes<br>②no<br>③I do not know
**Item 7:** Asymptomatic cases are also contagious	①yes<br>②no<br>③I do not know
**Item 8:** Only wearing multi-layer masks can prevent COVID-19.	①yes<br>②no<br>③I do not know
**Item 9:** Disposable surgical masks can be used repeatedly for a long time as long as they are not worn.	①yes<br>②no<br>③I do not know
**Item 10:** Chinese-manufactured vaccines are effective against the constantly mutating strain, such as Delta and Omicron.	①yes<br>②no<br>③I do not know
**Item 11:** People who have been vaccinated cannot be infected with COVID-19.	①yes<br>②no<br>③I do not know
**Item 12:** A safe social distance of more than one meter should be maintained in public places during the pandemic.	①yes<br>②no<br>③I do not know
**Item 13:** The "Dynamic-zero COVID-19 policy" adopted by China	①yes

means "zero infection."	②no<br>③I do not know
**Item 14:** People returning home from medium- and high-risk areas within 14 days only need to provide negative nucleic acid test results, and centralized or home quarantine is not necessary.	①yes<br>②no<br>③I do not know
**Item 15:** If there is an epidemic in your community, you can escape before lockdown.	①yes<br>②no<br>③I do not know
**Item 16:** If you have suspected symptoms related to COVID-19, you can take public transport to the hospital.	①yes<br>②no<br>③I do not know
**COVID-19 attitude items**	**Answers**
**Item 17:** Do you currently actively pay attention to the situation of COVID-19 at home and abroad?	①strongly disagree<br>②disagree<br>③general<br>④agree<br>⑤strongly agree
**Item 18:** Do you agree with the "Dynamic zero COVID-19 strategy" adopted by China?	①strongly disagree<br>②disagree<br>③general<br>④agree<br>⑤strongly agree
**Item 19:** Do you agree that China can successfully prevent infection from imported strains?	①strongly disagree<br>②disagree<br>③general<br>④agree<br>⑤strongly agree
**Item 20:** Do you agree that China can successfully cope with the domestic epidemic?	①strongly disagree<br>②disagree<br>③general<br>④agree<br>⑤strongly agree
**Item 21:** Do you agree that coping with COVID-19 requires the efforts of the whole society?	①strongly disagree<br>②disagree<br>③general<br>④agree<br>⑤strongly agree
**Item 22:** Do you agree that good vaccination coverage is effective in infection prevention and control?	①strongly disagree<br>②disagree<br>③general<br>④agree<br>⑤strongly agree
**Item 23:** Do you discriminate or ostracize COVID-19 patients or communities they live in?	①strongly disagree<br>②disagree<br>③general<br>④agree<br>⑤strongly agree
**COVID-19 practice items**	**Answers**
**Item 24:** Would you cooperate with national nucleic acid tests?	①never<br>②occasionally

	③sometimes<br>④often<br>⑤always
**Item 25:** Would you report to the infection prevention department after having contact with people infected with COVID-19 or experiencing suspected symptoms?	①never<br>②occasionally<br>③sometimes<br>④often<br>⑤always
**Item 26:** Would you cooperate with epidemiological investigations?	①never<br>②occasionally<br>③sometimes<br>④often<br>⑤always
**Item 27:** If you cough or sneeze, do you consciously cover your nose and mouth with a paper towel, handkerchief, or cloth?	①never<br>②occasionally<br>③sometimes<br>④often<br>⑤always
**Item 28:** Do you immediately disinfect the packages after collecting them?	①never<br>②occasionally<br>③sometimes<br>④often<br>⑤always
**Item 29:** Do you wash your hands after coming home?	①never<br>②occasionally<br>③sometimes<br>④often<br>⑤always
**Item 30:** When entering public places, such as restaurants, workplaces, and supermarkets, do you cooperate with anti-infection measures, including scanning health codes and checking temperature?	①never<br>②occasionally<br>③sometimes<br>④often<br>⑤always
**Item 31:** Do you actively participate in community infection prevention and control and provide suggestions for infection prevention and control?	①never<br>②occasionally<br>③sometimes<br>④often<br>⑤always
**Item 32:** Do you actively observe social distancing guidelines, such as maintaining a safe social distance and avoiding parties?	①never<br>②occasionally<br>③sometimes<br>④often<br>⑤always
**Item 33:** Do you avoid forwarding or spreading false information about COVID-19 during the pandemic?	①never<br>②occasionally<br>③sometimes<br>④often<br>⑤always
**Item 34:** Have you engaged in cyber-bullying of COVID-19 patients or close contacts?	①never<br>②occasionally

	③sometimes<br>④often<br>⑤always

Items 23 and 34 are reverse-scored

Supplementary Table 2. Participants' COVID-19 attitude Scores

Attitude items	lower than good (n = 473）	good and higher than good (n = 881）	Total (n = 1354）
Do you discriminate or ostracize COVID-19 patients or the communities in which they live?	3.07 ± 1.11	4.06 ± 1.16	3.71 ± 1.23
Are you currently actively paying attention to the COVID-19 situation at home and abroad?	3.51 ± 0.91	4.25 ± 0.70	3.99 ± 0.85
Do you agree that China can successfully prevent infection from imported strains?	3.69 ± 0.87	4.64 ± 0.57	4.31 ± 0.83
Do you agree with the "Dynamic zero COVID-19 strategy" adopted by China?	3.87 ± 0.80	4.73 ± 0.48	4.43 ± 0.73
Do you agree that China can successfully cope with the domestic epidemic?	3.95 ± 0.79	4.78 ± 0.44	4.49 ± 0.70
Do you agree that good vaccination coverage is effective in infection prevention and control?	4.08 ± 0.85	4.80 ± 0.42	4.55 ± 0.70
Do you agree that coping with COVID-19 requires the efforts of the whole society?	4.51 ± 0.78	4.96 ± 0.19	4.80 ± 0.54

Supplementary Table 3. Participants' COVID-19 practice Scores

Practice items	lower than good (n = 389）	good and higher than good (n = 965）	Total (n = 1354）
Do you immediately disinfect the packages after collecting them?	2.50 ± 1.13	3.83 ± 1.11	3.45 ± 1.27
Do you actively participate in community infection prevention and control and provide suggestions for infection prevention and control?	3.18 ± 1.13	4.17 ± 0.96	3.89 ± 1.10
Do you wash your hands after coming home?	3.44 ± 1.11	4.53 ± 0.69	4.21 ± 0.97
Have you engaged in cyber-bullying of COVID-19 patients or close contacts?	3.89 ± 1.53	4.36 ± 1.42	4.22 ± 1.47
Do you actively observe social distancing guidelines, such as maintaining a safe social distance and avoiding parties?	3.80 ± 0.95	4.67 ± 0.58	4.42 ± 0.81
Would you report to the infection prevention department after having contact with people infected with COVID-19 or experiencing suspected symptoms?	3.97 ± 1.01	4.75 ± 0.55	4.53 ± 0.80
If you cough or sneeze, do you consciously cover your nose and mouth with a paper towel, handkerchief, or cloth?	4.11 ± 0.96	4.79 ± 0.49	4.60 ± 0.73
Do you avoid forwarding or spreading false information about COVID-19 during the pandemic?	4.19 ± 1.09	4.84 ± 0.53	4.65 ± 0.79
Would you cooperate with epidemiological investigations?	4.31 ± 0.82	4.88 ± 0.38	4.71 ± 0.60
When entering public places, such as restaurants, workplaces, and supermarkets, do you cooperate with anti-infection measures, including scanning health codes and checking temperature?	4.33 ± 0.83	4.92 ± 0.28	4.75 ± 0.57
Would you cooperate with national nucleic acid tests?	4.46 ± 0.80	4.93 ± 0.26	4.80 ± 0.53

2 Supplementary Figures

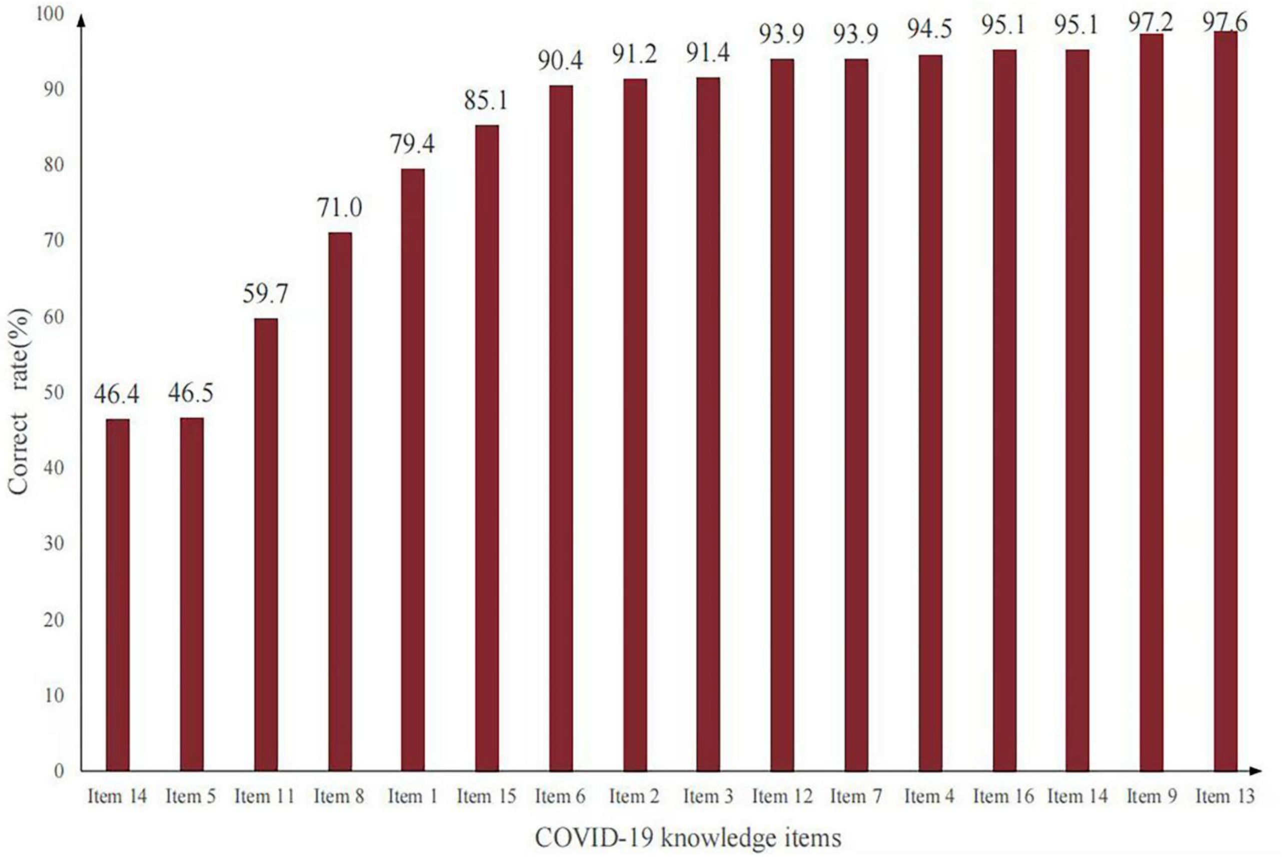

Supplementary Figure 1. Correctness rate of knowledge regarding COVID-2019 among participants.
